# Campbell title registrations to date – November 2023, and discontinued protocols

**DOI:** 10.1002/cl2.1375

**Published:** 2023-12-03

Details of new titles for systematic reviews or evidence and gap maps that have been accepted by the Editor of a Campbell Coordinating Group are published in each issue of the journal. If you would like to receive a copy of the approved title registration form, please send an email to the Managing Editor of the relevant Coordinating Group.

A list of discontinued protocols appears below these new titles. If you are interested to continue a project, please get in touch with the Managing Editor of the relevant Coordinating Group or email info@campbellcollaboration.org.

## BUSINESS AND MANAGEMENT

Gender diversity on corporate boards and the relationship to corporate financial performance: Is gender in corporate boards settings of any importance? A systematic review and meta‐analysis

Anders McIlquham‐Schmidt, Cédric Velghe

31 October 2023

## CHILDREN AND YOUNG PERSONS WELLBEING

Breakfast consumption, anthropometry, and nutrition‐related outcomes in adolescents from low‐ and middle‐income countries: A systematic review and meta‐analysis

Jordie Fischer, Jonathan Thomas, Kesso van Zutphen‐Küffer, Despo Ierodiakonou, Klaus Kraemer, Vanessa Garcia‐Larsen

13 November 2023

## CLIMATE SOLUTIONS

Role of behavioral and monetary interventions in reducing energy consumption in households: A ‘living’ systematic review

Jan Minx, Tarun Khanna, Max Callaghan, Klaas Miersch, Julian Elliot, Mark Andor

19 September 2023

## CRIME AND JUSTICE

Interventions to identify violence against women in high‐income countries: An evidence and gap map of impact evaluations

Charlotte Bell, Lorelei Hine, Lauren Hamilton, Elizabeth Watt, Elizabeth Eggins, Angela Higginson

20 November 2023

## EDUCATION

Identifying evidence‐based guidance for anti‐racism allyship in higher education: A scoping review

Marian Luctkar‐Flude, Laura Killam, Alexandra Hodson, Monica Larocque, Nikita Gupta, Amanda Ross‐White, Jane Tyerman

18 September 2023

Effective teacher preparation programs for improving academic achievement: A systematic review and meta‐analysis

Roisin Corcoran, Sarah Miller, Joanne O'Flaherty, Patrick Labelle

27 October 2023

## SOCIAL WELFARE

The effects of creative arts therapy on the treatment of alcohol and other drugs related problems: A mixed methods systematic review

Arianne Reis, Kaniz Fatema, Philip Hayward, Anna Kearns, Nicole Peel, Gilbert Whitton

12 October 2023

## DISCONTINUED PROTOCOLS

If you are interested to continue one of the projects below, please get in touch with the Managing Editor of the relevant Coordinating Group or email info@campbellcollaboration.org.

## CRIME AND JUSTICE

https://onlinelibrary.wiley.com/doi/10.1002/CL2.85

PROTOCOL: Mandatory arrest for misdemeanor domestic violence effects on repeat offending

Barak Ariel, Lawrence W. Sherman

## EDUCATION

https://onlinelibrary.wiley.com/doi/10.1002/CL2.163

PROTOCOL: Provision of information and communications technology (ICT) for improving academic achievement and school engagement in students aged 4–18

Kristin Liabo, Laurenz Langer, Antonia Simon, Kathy‐Ann Daniel‐Gittens, Alex Elwick, Janice Tripney

https://onlinelibrary.wiley.com/doi/10.1002/CL2.110

PROTOCOL: Education support services for improving school engagement and academic performance of children and adolescents with a chronic health condition

Michelle A. Tollit, Susan M. Sawyer, Savithiri Ratnapalan, Tony Barnett

https://onlinelibrary.wiley.com/doi/10.1002/CL2.197

PROTOCOL: Electronic mentoring to promote positive youth outcomes for young people under 25: A systematic review

Robyn M. O'Connor, David L. DuBois, Lucy Bowes

https://onlinelibrary.wiley.com/doi/10.1002/CL2.166

PROTOCOL: Merit pay programs for improving teacher retention, teacher satisfaction, and student achievement in primary and secondary education: A systematic review

Gary Ritter, Julie Trivitt, Leesa Foreman, Corey DeAngelis, George Denny

## INTERNATIONAL DEVELOPMENT

https://onlinelibrary.wiley.com/doi/10.1002/cl2.1186

PROTOCOL: The association between whole‐grain dietary intake and noncommunicable diseases: A systematic review and meta‐analysis

Wasim A. Iqbal, Gavin B. Stewart, Abigail Smith, Linda Errington, Chris J. Seal

https://onlinelibrary.wiley.com/doi/10.1002/CL2.84

PROTOCOL: Nutrition interventions and programs for reducing mortality and morbidity in pregnant and lactating women and women of reproductive age: A systematic review

Philippa Middl

https://onlinelibrary.wiley.com/doi/10.1002/CL2.171

PROTOCOL: Agronomic biofortification strategies to increase grain zinc concentrations for improved nutritional quality of wheat, maize and rice: A systematic review

Israel F. N. Domingos, Marcin Baranski, Carlo Leifert, Ismail Cakmak, Zed Rengel, Paul E. Bilsborrow, Gavin B. Stewart

https://onlinelibrary.wiley.com/doi/10.1002/CL2.138

PROTOCOL: Improving maternal, newborn and women's reproductive health in crisis settings

Primus Che Chi, Henrik Urdal, Odidika UJ Umeora, Johanne Sundby, Paul Spiegel, Declan Devane

https://onlinelibrary.wiley.com/doi/10.1002/CL2.183

PROTOCOL: The impacts of interventions for female economic empowerment at the community level on human development

Marcela Ibanez, Sarah Khan, Anna Minasyan, Soham Sahoo, Pooja Balasubramanian

https://onlinelibrary.wiley.com/doi/10.1002/CL2.154

PROTOCOL: Peer‐based interventions for reducing morbidity and mortality in HIV‐positive women

J. Petkovic, M. Doull, A. M. O'Connor, J. Aweya, M. Yoganathan, V. Welch, G. A. Wells, P. Tugwell

https://onlinelibrary.wiley.com/doi/10.1002/CL2.188

PROTOCOL: Community‐led total sanitation in rural areas of low‐ and middle‐income countries: A systematic review of evidence on effects and influencing factors

Josef Novotný, Jiří Hasman, Martin Lepič, Vít Bořil

https://onlinelibrary.wiley.com/doi/10.1002/CL2.140

PROTOCOL: Access to electricity for improving health, education and welfare in low‐ and middle‐income countries

Kavita Mathur, Sandy Oliver, Janice Tripney

https://onlinelibrary.wiley.com/doi/10.1002/CL2.157

PROTOCOL: Saving promotion interventions for improving saving behaviour and reducing poverty in low‐ and middle‐income countries

Janina Isabel Steinert, Ani Movsisyan, Juliane Zenker, Ute Filipiak, Yulia Shenderovich

https://onlinelibrary.wiley.com/doi/10.1002/CL2.172

PROTOCOL: The effect of interventions for women's empowerment on children's health and education

Sebastian Vollmer, Sarah Khan, Le Thi Ngoc Tu, Atika Pasha, Soham Sahoo

https://onlinelibrary.wiley.com/doi/10.1002/CL2.200

PROTOCOL: The effects of road infrastructure, and transport and logistics services interventions on women's participation in informal and formal labour markets in low‐ and middle‐income countries

Manisha Gupta, Souvik Bandyopadhyay, Meerambika Mahapatro, Shreya Jha

## SOCIAL WELFARE

https://onlinelibrary.wiley.com/doi/10.1002/cl2.1259

PROTOCOL: Effects of virtual reality exposure therapy versus in vivo exposure in treating social anxiety disorder in adults: A systematic review and meta‐analysis

Martin Polak, Norbert Tanzer, Per Carlbring

https://onlinelibrary.wiley.com/doi/10.1002/CL2.109

PROTOCOL: The therapeutic alliance and psychotherapy outcomes for young adults aged 18 to 34

Stacy J. Green, Julia H. Littell, Karianne Hammerstrøm, Emily Tanner‐Smith, Jessica Schaffner Wilen

https://onlinelibrary.wiley.com/doi/10.1002/CL2.56

PROTOCOL: Cognitive‐behavioural therapy for parents who have physically abused their children

Mogens N. Christoffersen, Jacqueline Corcoran, Diane DePanfilis, Claire Daining

